# Shock Wave Therapy in the Treatment of Erection Dysfunction: How to Define Clinical Outcomes? A Comparison Between Penile Doppler Ultrasound and a New Visual Erection Hardness Score (V-EHS) During a Blinded, Sham-Controlled Trial

**DOI:** 10.1590/S1677-5538.IBJU.2024.9927

**Published:** 2025-01-10

**Authors:** Mathias Ferreira Schuh, Rodrigo Ribeiro Vieiralves, Luciano Alves Favorito

**Affiliations:** 1 Lagoa Federal Hospital Departamento de Urologia Rio de Janeiro RJ Brasil Departamento de Urologia, Lagoa Federal Hospital, Rio de Janeiro, RJ, Brasil;; 2 Universidade do Estado do Rio de Janeiro Unidade de Pesquisa Urogenital Rio de Janeiro RJ Brasil Unidade de Pesquisa Urogenital, Universidade do Estado do Rio de Janeiro, UERJ, Rio de Janeiro, RJ, Brasil

**Keywords:** Extracorporeal Shockwave Therapy, Erectile Dysfunction, Ultrasonography, Doppler

## Abstract

**Background:**

In the last decade, several studies have proven the effectiveness of low-intensity shock waves (LI-ESWT), but with several factors that make it difficult to carry out systematic reviews.

**Aim:**

To demonstrate the effectiveness of LI-ESWT and define the best tool for routine clinical assessment of erectile dysfunction.

**Materials and Methods:**

Twenty-one participants with purely vasculogenic erectile dysfunction were selected and randomized to LI-ESWT or placebo. All patients underwent evaluation with The International Index of Erectile Function (IIEF-5), V-EHS (new visual scale), and standardized penile doppler ultrasound before and after shock wave therapy.

**Outcomes:**

LI-ESWT has proven effective in the treatment of moderate erectile dysfunction, and the new V-EHS has demonstrated greater accuracy than Doppler in the diagnosis and follow-up of erectile dysfunction.

**Results:**

Using the IIEF-5 as a control tool, we observed a clinical response after 1 month, with a greater increase in the shock wave therapy arm of +3.21 points compared to + 0.57 in the sham group. At six months, the treated group showed a mean increase of 4.71 points compared to baseline (p = 0.006), while those who received sham therapy had a decrease (case = +4.71 points vs. sham control = −1.0, p = 0.006). Based on this observed difference, we performed a comparative analysis between the V-EHS and penile doppler ultrasound to observe whether the test results corroborated the IIEF-5 findings. The correlation between V-EHS and IIEF-5 in the therapy group in the pre-therapy period was strong (r = 0.816, p < 0.001), and at 6 months it increased to very strong (r = 0.928, p < 0.001). Penile Doppler ultrasound did not show the same correlation strength with IIEF-5, presenting a moderate correlation at 6 months (Pearson correlation score = 0.540), as also demonstrated in the ROC curve through the V-EHS AUC = 0.963 (p = 0.001) vs. Doppler AUC = 0.713 (p = 0,290).

**Strengths and Limitations:**

The main strengths of the present study are the blinded, randomized, placebo-controlled clinical trial and the comparison between penile Doppler and a new visual classification for erection hardness score. The limitations are the number of patients and the short follow-up.

**Conclusions:**

LI-ESWT has proven effective in the treatment of moderate vasculogenic erectile dysfunction, with optimal results at 6 months. The new V-EHS offers a simple, reliable and reproducible assessment of erectile function.

## INTRODUCTION

Erectile dysfunction (ED) is a common condition that affects approximately 18 million men in the United States. It is characterized by the persistent inability to achieve or maintain an erection sufficient for satisfactory sexual activity, which significantly affects the quality of life (1). Among the current treatment options, low-intensity extracorporeal shock wave therapy (LI-ESWT) has shown good results. Many studies and international guidelines recommend it as an extra treatment for men with mild to moderate vasculogenic erectile dysfunction (2, 3).

This new therapy emerged with the hope of being the only modality capable of acting directly on the pathophysiology of ED, offering remodeling of the erectile tissue and, therefore, some degree of recovery of erectile function by promoting neovascularization, which has a positive effect on penile hemodynamics (2-4). However, like all new technologies especially those involving highly technical aspects such as new devices and different types of energy, along with physical aspects that are not familiar to the urologist's routine, this therapy requires time and continuous verification to gain the trust of doctors necessary for recommending it (4).

In this scenario, finding tools that allow clinicians to ensure the results obtained from this new treatment modality can be considered a turning point in the certification of this technology and in the safety of the method's indication. Since the validation of the Erection Hardness Score by Dr. John Mulhall and colleagues in 2007, this functional score has been extensively used in clinical practice (5). However, the lack of standardization in studies aimed at evaluating the improvement of erectile dysfunction after shock wave therapy is notorious. The established use of the IIEF-5, in addition to the EHS and penile Doppler ultrasound, has been conducted without standardization to determine which parameters demonstrate the most accurate results (6-8). Despite the recognition of both the EHS and penile Doppler ultrasound as established tools in the evaluation of patients with erectile dysfunction, the absence of a visual scale for the EHS and the lack of standardization in penile Doppler protocols complicates the interpretation of results. Recent efforts have been published in the sexual medicine literature to address this need for standardization (9-12).

In the present study, we observed the effects of shock wave therapy using the International Index of Sexual Function in its summarized version (IIEF-5) as a control parameter to verify the correlation of the results obtained through penile Doppler and an evaluation of a new visual scale for the Erection Hardness Score (visual erection hardness score – V-EHS). Our hypothesis is that the LI-ESWT improves mild and moderate ED. We aim to prospectively evaluate the efficacy of low-intensity shockwave therapy in patients with mild and moderate ED.

## MATERIALS AND METHODS

The present study was approved according to the ethical standards of the hospital's institutional committee on experimentation with human beings. We implemented a 2-arm stratified single-blinded randomized controlled clinical trial to determine the impact of sham versus LI-SWT on erectile function (IRB: 72872821.5.0000.5259). We confirm that all methods used in this paper were carried out in accordance with relevant guidelines and regulation in compliance to the declaration of Helsinki.

**Figure 1:**
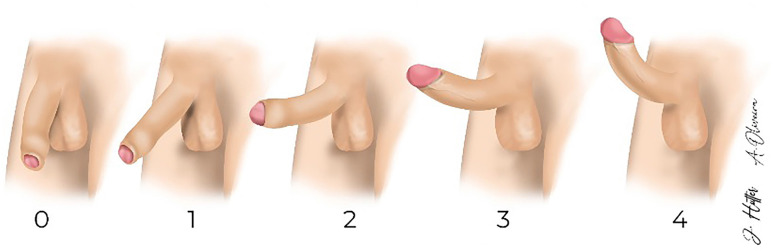
The figure shows the Visual Erection Hardness Score (V-EHS). This score is derived from the original Erection Hardness Score (5) but some modifications are incorporated: 1) The patient does not subjectively score; 2) It presents a new image, facilitating the perception and differentiation between the stages; 3) The scale itself, as we see above, is differentiated according to the axial resistance that the penis supports, which is functionally and directly related to the penetrative capacity and 4) It allows standardizing the erection test and the time of the re-dose (which should be done if a consistently hard erection (>3) is not obtained). In the figure we can observe: 0: Penis does not enlarge; 1: Penis is larger but not hard; 2: The penis is hard, but not hard enough to resist an axial force - it bends under a manual pulling force = not consistently hard erection; 3: Penis is hard, not completely hard, but resists an axial force - does not bends under a manual pulling force = consistently hard erection; 4: Penis is completely hard and fully rigid.

Data were collected between June 2022 and March 2024. The initial selection of patients was according to the baseline clinical complaint of erectile dysfunction and the presence of moderate erectile dysfunction based on the validated International Index of Erectile Function questionnaire (IIEF-5 - 8 to 21) in use of tadalafil 5 mg daily. All pre-selected patients were referred for the Visual Erection Hardness Score (V-EHS), which is derived from the original EHS (5) with the inclusion of some modifications that are described in the Figure-1 and dynamic Doppler ultrasonography of the penis in order to confirm the presence of vasculogenic erectile dysfunction.

The criteria used in the positive determination of vasculogenic ED were: clinical history with cardiovascular risk factors and penile doppler ultrasound with peak systolic velocity (PSV) < 30 cm/s, end-diastolic velocity (EDV) > 5 cm/s, or cavernous resistance index (RI) <0,9. The V-EHS and penile doppler ultrasound evaluations were performed during a pharmaco-induced erection test by recording the time after intracavernous injection of trimix - 0.3 mL (prostaglandin 20 mcg/mL + phentolamine 4 mg/mL + papaverine 25 mg/mL) using as a basis for a re-dose the visual rigidity score (V-EHS) (Figure-1). If, after 20 minutes, the patient did not achieve a consistently hard erection (V-EHS = 3), a second dose was administered with the same concentration and volume.

**Figure 2:**
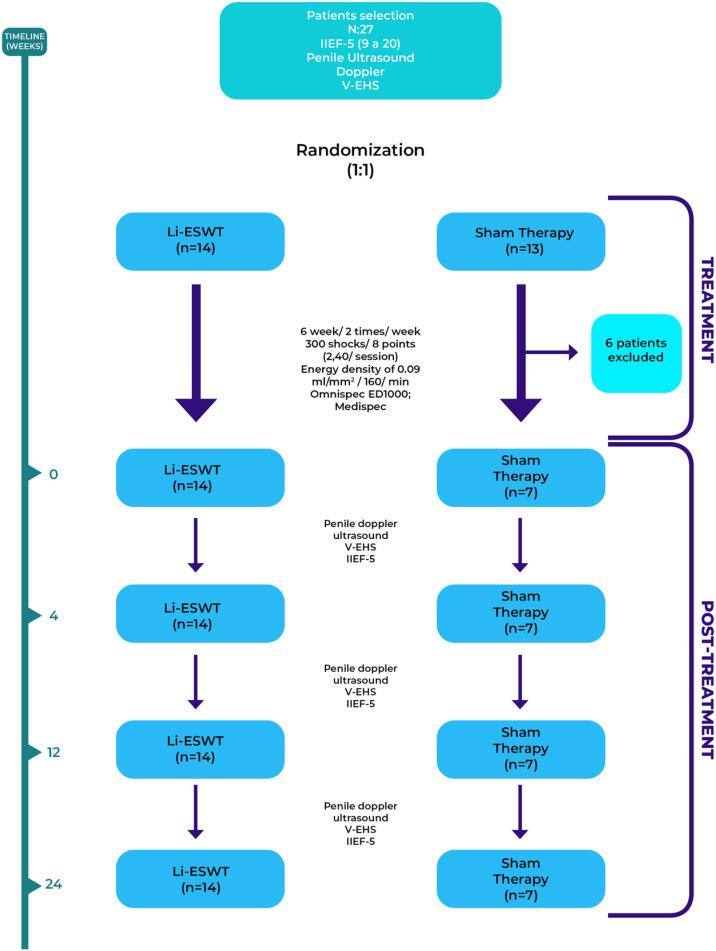
The figure shows the protocol used during the present study.

Patients were excluded in cases of: (1) unstable psychiatric condition, (2) previous history of e penile/urethral surgery, (3) proven hypogonadism and (4) severe erectile dysfunction. The protocol of the present study is shown in Figure-2. Patients were randomized in a ratio of 1:1 into two groups: case-low intensity shock wave therapy (n = 14) or control (sham group) (n = 7).

Our LI-ESWT protocol was performed in 12 sessions, twice a week, for 6 weeks. We used the electro-hydraulic generating unit with a focal shock wave source (Omnispec ED1000; Medispec, Germantown, MD, USA). For the shock wave therapy session, the patient remained lying down in a supine position, the penis was manually stretched, and a standard commercial gel normally used for ultrasound was applied to the entire area of interest. The shock waves were distributed through the application probe to 8 sites: the distal, middle, and proximal penile shafts (both sides) and to the crura bilaterally, considering the final point of interest to be the corpora cavernosa and not only the penile shaft. Sessions consisted of 300 shocks for each treatment site (2,400/session) at an energy density of 0.09 mJ/mm2 and a frequency of 160/min. For the sham group, the sessions occurred in a similar manner, with the application probe being applied in an identical manner and the sound reproduced by a speaker located attached to the generator. Patient monitoring was performed in the outpatient clinic at 1, 3, and 6 months with IIEF-5, penile Doppler ultrasound, and V-EHS.

The erection tests, the V-EHS assessment, and penile Doppler ultrasound were performed by the same examiner. Treatment success was defined as an improvement of 4 points or more in the IIEF-5, as it had greater clinical significance. All patients continued to use tadalafil 5 mg throughout the study protocol.

### Statistical analysis

The statistical analyses were performed using IBM SPSS, version 20. Data were presented in tables of means and standard deviations. A student's t-test for independent samples was used to statistically evaluate the differences between the Case and Sham groups for the quantitative interval variables. To evaluate the differences over time (pre, 1 month, 3 months, and 6 months) of the IIEF-5 and V-EHS scales, the nonparametric Friedman test was used, followed by Dunn's paired comparison tests. The differences between the Case and Sham groups at each time point of the IEEF-5 and V-EHS scales were verified using the Mann-Whitney test. To statistically evaluate the differences between groups, between assessments (pre, 1 month, 3 months, and 6 months), and the interaction between groups and assessments for the measurements of peak velocity of the right (r-PSV) and left (l-PSV) cavernous artery, the ANOVA technique for repeated measures was used, with a within-subject factor (repeated measure) and a between-subject factor. The within-subject factor was represented by the assessments, and the between-subject factor was represented by the groups. The significance level used as a criterion for acceptance or rejection in the statistical tests was 5% (p < 0.05).

The Pearson Correlation Index was used to determine the value of the correlation coefficient. The value of the correlation coefficient can range from - 1 to +1. The closer to −1, the stronger the negative correlation between the variables (negative correlation indicates that the higher the values of one variable, the lower the values of the other variable tend to be). The closer to +1, the stronger the positive correlation between the variables (positive correlation indicates that the higher the values of one variable, the higher the values of the other variable tend to be). Coefficient values close to 0 (zero) indicate an absence of correlation.

For ROC curve, area under the curve (AUCs) <0.5, between 0.5 and 0.7, between 0.7 and 0.8, and >0.8, the test was considered worthless, acceptable, good, or excellent, respectively. DeLong's empirical method was used to compare the AUC without a pairwise approach. All tests were 2-sided, and statistical significance was considered at a P value<0.05.

## RESULTS

A total of 21 patients completed the protocol with 6 months of follow-up (6 patients in the sham group were excluded after initial recruitment because they missed more than one therapy session). The mean age of patients was 62.71 ± 9.38 years, and cardiovascular risk factors were common among participants in both groups (Table-1). The most frequent comorbidities were systemic arterial hypertension (57.1%), followed by type 2 diabetes mellitus (23.8%).

All data regarding IIEF-5 parameters, penile hemodynamic findings (PSV, EDF, and RI), and V-EHS pre-treatment at 1, 3, and 6 months are described in Table-1. The diagnosis of arterial insufficiency was made in all cases, with 3 patients in the treated group and 3 patients in the sham group requiring a re-dose of trimix to achieve their best erection quality.

Before the sessions of shock wave therapy, the group that would undergo treatment presented IIEF-5 of 14.29 ± 3.173 points and the control group (sham) 12.57 ± 2.507 points. After 1 month, the treated group presented IIEF-5 of 17.50 ± 6,430 and the control group (sham) 13.14 ± 4.670 (p=0.149). After 3 months, the treated group presented IIEF-5 of 18.86 ± 6.037 and the control group (sham) (12.43 ± 4.467) p-value = 0.020. Finally, at 6 months after low-intensity shock wave therapy, after 3 months of treatment, the treated group presented IIEF-5 of 19 ± 5.657 and the control group (sham) 11.57 ± 2.760 p-value = 0.006.

Before starting LI-ESWT, in the case group, there was a strong positive correlation between IIEF-5 and V-EHS (r =0.816, p<0.001), indicating that even before the procedure, erectile function was strongly associated with the new visual erectile function score. In this same period, the correlations between IIEF-5 and the systolic velocities of the right (r =0.415, p=0.140) and left (r =0.217, p=0.455) cavernous arteries were not statistically significant.

After 1 month of LI-ESWT, in the treatment group, the correlation between IIEF-5 and V-EHS increased to very strong (r=0.945, p<0.001). The correlation between IIEF-5 and the right cavernous artery was weak (r=0.436, p=0.119), and between IIEF-5 and the left cavernous artery was weak (r=0.354, p=0.215), both of which were not statistically significant. In the control group, the correlation between IIEF-5 and V-EHS was also strong and significant (r=0.872, p=0.011), but the correlations with the right (r=0.348, p=0.445) and left (r=0.116, p=0.805) cavernous arteries were not significant.

At 3 months, in the treatment group, the correlation between IIEF-5 and V-EHS remained very strong (r=0.970, p<0.001). The correlations between IIEF-5 and the systolic velocities of the right (r=0.307, p=0.285) and left (r=0.476, p=0.085) cavernous arteries were again not statistically significant. In the control group, the correlation between IIEF-5 and V-EHS remained strong (r=0.868, p=0.011), and the correlations with the right (r=-0.295, p=0.521) and left (r=-0.228, p=0.623) cavernous arteries remained non-significant.

At 6-month final follow-up, in the treatment group, the correlation between IIEF-5 and V-EHS remained very strong (r =0.928, p<0.001). The correlation between IIEF-5 and the right cavernous artery was moderate (r =0.510, p =0.062), whereas the correlation between IIEF-5 and the left cavernous artery was weak (r=0.404, p=0.152). In the control (sham) group, the correlation between IIEF-5 and EHS remained strong and significant (r=0.825, p=0.022), but the correlations with the right (r =0.124, p=0.791) and left (r=-0.331, p=0.468) cavernous arteries were not significant.

**Figure 3:**
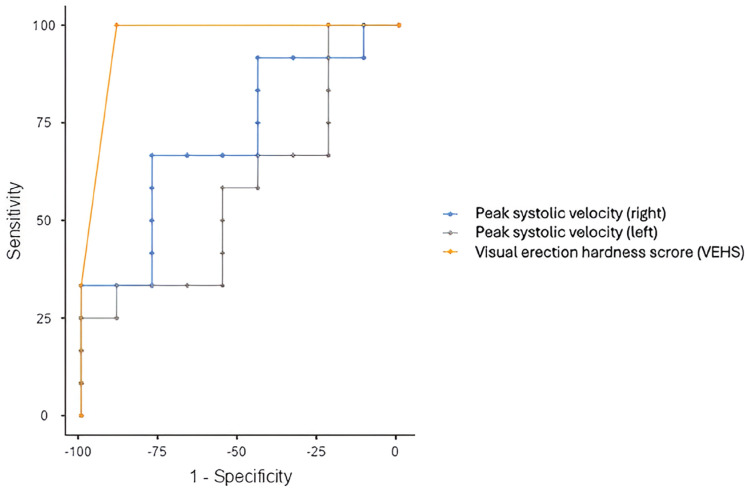
The figure shows the ROC Curves for Visual Erection Hardness Score (V-EHS) and peak systolic velocity (PSV) based on clinical improvement in erectile function with V-EHS showing a sensitivity of 100% and a specificity of 88.89% vs. 66.67% sensitivity and 77% specificity for penile Doppler USG.

The ROC curves for V-EHS and PSV based on clinical improvement in erectile function are shown in Figure-3. The AUCs for right and left PSV and V-EHS to discriminate clinical improvement from ED (4 or more points improvement in IIEF-5) were 0.713 (p=0.035), 0.574 (p=0.290), and 0.963 (p=0.001), respectively. V-EHS was rated as excellent, right PSV as good, and left PSV as acceptable in discriminating clinical improvement. Pairwise comparison of ROC curves showed a statistically significant difference between V-EHS and Doppler PSV (p=0.0301), with V-EHS showing a sensitivity of 100% and a specificity of 88.89% vs. 66.67% sensitivity and 77% specificity for penile Doppler USG.

## DISCUSSION

The present study demonstrated a significant improvement in erectile function in patients treated with LI-ESWT for mild to moderate vasculogenic erectile dysfunction. This improvement was evidenced by a mean increase of 4.71 points in IIEF-5 six months after treatment, compared to a decrease of −1.0 points in the placebo group, as shown in previous literature demonstrating the short-term clinical efficacy of low-intensity shock waves in cases of mild to moderate vasculogenic erectile dysfunction (11, 12). A previous review shows that LI-ESWT has the potential to promote tissue remodeling through neovascularization and partial recovery of erectile function (13). Although the efficacy of LI-ESWT is promising, the review also highlights a lack of standardization in terms of treatment protocols, including the applied energy, number of sessions, and application sites, factors that can influence the observed outcomes. Despite being an innovative therapy, LI-ESWT still lacks robust and higher-quality studies to consolidate its clinical indication (14-20). The present study employed a standardized protocol with 12 treatment sessions over six weeks, which may explain the consistency of the short-term results.

We observed a strong correlation between V-EHS and IIEF-5 in the shockwave group. On the other hand, penile Doppler only showed a moderate correlation with IIEF-5 over the same period, suggesting that V-EHS may be a more reliable predictor of erectile function in the context of ED therapies. Considering the findings described, the V-EHS presented greater accuracy (sensitivity and specificity) when compared to penile Doppler (PSV, EDF, and RI) in predicting the degree of erectile dysfunction and the presence of clinical improvement (or refractory ED) after low-intensity shock wave therapy.

These findings are consistent with a previous analysis (21-24), which compared EHS with penile Doppler in a study of patients treated with non-surgical therapy for ED. The study showed that EHS has predictive value similar to or even greater than Doppler in identifying patients with refractory ED, defined as failure to respond to non-invasive treatments such as sildenafil or alprostadil therapy. In our study, the AUCs for predicting clinical improvement in ED was higher for V-EHS (AUC=0.963) compared to Doppler, which corroborates previous findings (24).

We know that Penile Doppler ultrasound is widely regarded as a valuable tool for assessing penile hemodynamics, but its clinical utility has been questioned in some contexts. Our study demonstrated that penile Doppler did not show a high correlation with clinical outcomes, as indicated by the low correlation coefficients with IIEF-5 after six months of treatment. This raises questions about the practical applicability of penile Doppler in certain therapeutic contexts, particularly in non-invasive treatments such as LI-ESWT, promoting the healthy question of whether the assessment of erection rigidity is not a more accurate form of assessment because, in addition to inferring the vascular factor, it also assesses the expansion of the tunica albuginea and possible geometric alterations that cause penile instability, such as EHS and now the new V-EHS (25, 26).

This debate is highlighted by studies like that of Morgado et al., which point to the lack of additional prognostic value provided by Doppler compared to the simpler intracavernosal injection test (27). While Doppler can provide detailed information about penile blood flow, it is often seen as overly complex and time-consuming, with little added value over a pharmaco-induced erection test in predicting treatment response to sildenafil or other oral therapies for ED. Additionally, the variability in Doppler protocols, such as the use of different vasoactive agents, doses, and time intervals, can result in false diagnoses, as observed in other studies, which report false-positive diagnosis rates of up to 47% for venous-occlusive dysfunction.

In contrast to penile Doppler, V-EHS is a simple and practical tool that can be easily applied by clinicians during a pharmacologically induced erection assessment. As the EHS has been validated in several studies, such as that of Mulhall et al. (5), which demonstrated that it is highly responsive and correlates well with other measures of erectile function, such as IIEF, we think that V-EHS may assume a very important practical parameter. Mulhall's study also highlights the ease of use of EHS in clinical trials, being a direct and reliable measure of penile rigidity without the need for specialized equipment or advanced technical skills exactly as the new V-EHS (5). Furthermore, unlike even the subjective evaluation by the patient through EHS, the new V-EHS is carried out entirely by the examiner himself, during the erection test, without the need for the patient's own perception.

The results presented here further reinforce the utility of V-EHS, suggesting that it may be an adequate substitute for penile Doppler in many clinical situations, particularly in the evaluation of patients undergoing therapies for erectile dysfunction, not only LI-ESWT. The simplicity and reproducibility of V-EHS, combined with its strong correlation with IIEF-5, make it a valuable tool for clinical practice, especially in resource-limited settings.

Although our results are encouraging, both regarding the efficacy of LI-ESWT and the use of V-EHS as an assessment tool, the lack of standardization across studies is a recurring issue. As highlighted before, there is an urgent need for greater standardization in terms of treatment protocols and evaluation methods so that clinical outcomes can be comparable and replicable. Future studies should focus on expanding sample sizes and standardizing treatment parameters, such as the LI-ESWT energy dose, number of sessions, and the intervals between them, as well as defining consistent protocols for evaluating outcomes with V-EHS. With the implementation of these measures and through the use of penile rigidometers, it will be possible to obtain more accurate results and further validate the preliminary results of this study, in addition to consolidating LI-ESWT as first-line therapy for moderate vasculogenic ED.

The present paper has some limitations: The small sample size limits the generalizability of the findings, as acceptance of LI-ESWT and the relatively short follow-up.

## CONCLUSIONS

In the present study, low-intensity shockwave therapy was effective in the treatment of mild to moderate vasculogenic erectile dysfunction, with results observed from 1 month and optimized up to 6 months. The use of the new visual erection hardness score provides a simple, reliable, and reproducible assessment of erectile function and is therefore also a practical tool that allows the standardization of drug-induced erection testing.
